# A case of adult eosinophilic oesophagitis

**DOI:** 10.1186/2045-7022-1-S1-P90

**Published:** 2011-08-12

**Authors:** Isabel Skypala, Stephen Till

**Affiliations:** 1 Royal Brompton & Harefield NHS Trust, Rehabilitation & Therapies, London, UK; 2 Imperial College, London, Allergy and Clinical Immunology, London, UK

## Background

Adults with Eosinophilic Oesophagitis (EO) often present with a history of dysphagia and food impaction. Many are also atopic and sensitisation to foods is common; 81% of adults with EO in one study were sensitised to one or more food or inhalant allergen, and 50% had positive skin prick tests to one or more foods. Exclusion of foods has been reported to improve clinical symptoms; 94% of adults improved on a 6 food elimination diet, and in children, the use of an elemental formula has been proven to be effective.

## Method

We describe an adult male patient aged 30 years with a diagnosis of EO. Skin prick testing with fresh foods and specific IgE blood tests (Phadia ImmunoCAP) revealed sensitisation to a number of foods. Following an acute episode of dysphagia requiring dilatation, the patient commenced a complete exclusion diet. Nutritional support was maintained solely through the consumption of a nutritionally complete liquid elemental diet (Elemental 028™). After six weeks, one new food was introduced every 2-3 days.

## Results

The patient was sensitised to birch and grass pollen and a wide variety of foods including potatoes, carrots, broccoli, lettuce, spinach, beer, wine, grapes, apple juice, mango, peanut, mustard, marmite, rice, wheat, barley, yeast and the peach lipid transfer protein allergen Pru p 3. Following the 6-week elemental diet, to date the following foods have been successfully re-introduced into the diet; rice, fish, onions, eggs, olives, lamb, corn, barley, milk, vodka, red wine, champagne, barley, peas, pak choi, pork, turkey. Soy and grapes, both previously eaten regularly caused immediate symptoms upon re-introduction.

## Conclusion

This case demonstrates that in the face of multiple sensitisation to foods, an elemental diet followed by planned and careful staged re-introduction of foods may be effective in establishing which foods can be safely be consumed.

